# Tapping Into Rate Flexibility: Musical Training Facilitates Synchronization Around Spontaneous Production Rates

**DOI:** 10.3389/fpsyg.2018.00458

**Published:** 2018-04-06

**Authors:** Rebecca Scheurich, Anna Zamm, Caroline Palmer

**Affiliations:** Sequence Production Laboratory, Department of Psychology, McGill University, Montreal, QC, Canada

**Keywords:** musical expertise, spontaneous rates, temporal flexibility, synchronization, motor skill

## Abstract

The ability to flexibly adapt one’s behavior is critical for social tasks such as speech and music performance, in which individuals must coordinate the timing of their actions with others. Natural movement frequencies, also called spontaneous rates, constrain synchronization accuracy between partners during duet music performance, whereas musical training enhances synchronization accuracy. We investigated the combined influences of these factors on the flexibility with which individuals can synchronize their actions with sequences at different rates. First, we developed a novel musical task capable of measuring spontaneous rates in both musicians and non-musicians in which participants tapped the rhythm of a familiar melody while hearing the corresponding melody tones. The novel task was validated by similar measures of spontaneous rates generated by piano performance and by the tapping task from the same pianists. We then implemented the novel task with musicians and non-musicians as they synchronized tapping of a familiar melody with a metronome at their spontaneous rates, and at rates proportionally slower and faster than their spontaneous rates. Musicians synchronized more flexibly across rates than non-musicians, indicated by greater synchronization accuracy. Additionally, musicians showed greater engagement of error correction mechanisms than non-musicians. Finally, differences in flexibility were characterized by more recurrent (repetitive) and patterned synchronization in non-musicians, indicative of greater temporal rigidity.

## Introduction

Auditory-motor synchronization occurs when individuals coordinate their actions in time with external auditory events, as in conversational speech or joint music-making. Music performance is an ideal model for the study of flexibility in auditory-motor synchronization. Musical sequences are typically produced at a wide range of rates, and musicians are expected to flexibly change their production rates to achieve precise synchronization with one another (Palmer, 1997; Repp, 2005). Musical synchronization is not restricted to highly trained individuals, but also occurs in individuals with little to no musical training. For example, individuals without musical training can clap along with a musical beat at a concert. Several studies have examined differences in auditory-motor synchronization accuracy between trained and untrained individuals (Aschersleben, 2002; Repp and Doggett, 2007; Chen et al., 2008; Repp, 2010). Little is known about how extensive training alters the flexibility with which individuals coordinate their actions at different rates. We investigate here the underlying variables that influence rate flexibility across musical skill levels.

A dynamical systems perspective proposes that biological and physical systems have internal rhythms or oscillators that entrain or couple with quasi-periodic rhythms in the environment; synchronization occurs via changes in the intrinsic or natural frequency (rate) and the relative phase of these internal oscillators (Strogatz and Stewart, 1993; Large and Jones, 1999). Natural frequencies in rhythmic (periodic) tasks such as walking, speaking, and performing music have been measured in terms of the rates at which individuals naturally or spontaneously produce rhythmic sequences in the absence of external cues (Murray et al., 1964; Loehr and Palmer, 2011; Zamm et al., 2015, 2016). Spontaneous rates measured in isochronous (regular) finger-tapping tasks, usually collected in the absence of auditory feedback, are referred to as spontaneous motor tempi (SMT) (Drake et al., 2000a,b; McAuley et al., 2006). SMT reflect biases toward particular rates which change from faster in early childhood to slower in adulthood, (McAuley et al., 2006), and are slower in musicians than in non-musicians (Drake et al., 2000a). Spontaneous rates at which musicians perform naturally (in the presence of auditory feedback), referred to as spontaneous production rates (SPRs), similarly reflect biases toward performing at a particular rate, and have been proposed to represent natural frequencies of underlying oscillations that place constraints on synchronization accuracy. Partners who have similar natural frequencies, reflected by SPRs of solo performance, are more synchronous in duet performance than partners who have different SPRs (Loehr and Palmer, 2011; Palmer et al., 2013; Zamm et al., 2015, 2016), consistent with predictions of a dynamical system in which natural frequencies that are more similar couple with each other more strongly.

The natural frequency represented by an individual’s spontaneous rate may act as an attractor, or state that requires less energy expenditure and toward which a system’s behavior will converge over time (Strogatz and Stewart, 1993). According to this perspective, individuals should be pulled toward their spontaneous rates during tasks in which they are required to move at other rates. When the difference between an individual’s spontaneous rate and the external rate is too great, coupling between the individual’s internal oscillations and the external rate cannot occur (Strogatz and Stewart, 1993). Much research that has investigated spontaneous rates as attractors has focused on skilled individuals such as musicians (Loehr and Palmer, 2011; Zamm et al., 2015, 2016) who receive intensive training that is assumed to enhance their flexibility to perform at a large range of rates (MacKay, 1982). Untrained individuals might be expected to show greater constraints of an attractor on synchronization due to less flexibility in coordinating actions across a range of rates (Drake et al., 2000a,b). Thus, it is unknown whether spontaneous rates act as an attractor frequency that is stronger for less skilled (non-musicians) compared with more skilled individuals (musicians).

Different perspectives have been offered for the mechanisms that contribute to SPRs. In one perspective, spontaneous rates may be driven primarily by central timing mechanisms such as central pattern generators (Latash, 1992). Another perspective suggests that spontaneous rates arise primarily from peripheral (anatomical and biomechanical) properties of the body (Goodman et al., 2000). Investigations into contributions of central and peripheral mechanisms to SPRs have shown mixed results. Whereas spontaneous rates appear to be consistent across similar limbs in music performance (Zamm et al., 2015, 2016), evidence also suggests that the joints at which oscillatory movements are initiated influence spontaneous rates (Peckel et al., 2014). For example, leg swinging occurs at a slower frequency when initiated from the hip than from the knee (Peckel et al., 2014). Despite mixed results about contributions to spontaneous rates, synchronization-continuation tasks have shown that participants tend to drift back over time toward their spontaneous rates when initially cued to perform at other rates (Yu et al., 2003; McAuley et al., 2006).

Comparisons of musicians and non-musicians in tapping tasks suggest that synchronization accuracy is influenced both by musical training and spontaneous rates (Drake et al., 2000a,b). Drake et al. (2000a) compared the SMT of children and adults, both musically trained and untrained, as well as their synchronization with different auditory sequences. Musicians successfully synchronized more often than non-musicians across age groups, particularly for isochronous (regular intervals) and rhythmic sequences. Participants with slower SMT tended to synchronize with higher hierarchical levels (slower rates); this correlation was stronger for non-musicians than for musicians, suggesting that non-musicians were less able to synchronize with rates less similar to their SMT than musicians. These findings are consistent with those from Drake et al. (2000b), who showed that musicians were better able than non-musicians to synchronize their tapping with a musical excerpt at both higher and lower hierarchical levels of the meter than the level with which they naturally synchronized. Overall, musical training and spontaneous rates show different effects on synchronization such that musical training enhances flexibility across rates, whereas spontaneous rates cause a bias in synchronization with an attraction toward a natural frequency.

## Current Research

The current research examines the influences of spontaneous rates and musical training on rate flexibility in a synchronization task. To date, no studies have directly compared musicians’ and non-musicians’ synchronization accuracy in music production tasks, for the obvious reason that non-musicians cannot perform the same musical tasks as musicians. To compare synchronization abilities of musicians and non-musicians, we developed a novel musical task capable of measuring SPRs that could be implemented with individuals with limited or no musical expertise. Experiment 1 validated the novel musical tapping task by comparing pianists’ performances while tapping melody rhythms with normal piano performances of the same melodies. If the novel task elicits an experience similar to music performance, then SPRs across tasks should be similar. We also investigated contributions of peripheral mechanisms to SPRs by measuring pianists’ hand sizes (Yu et al., 2013). If peripheral mechanisms contribute to SPRs, then pianists with larger hands might be expected to show slower SPRs (Goodman et al., 2000).

Experiment 2 implemented the novel task with musician and non-musician participants to investigate the influences of spontaneous rates and musical training on rate flexibility of synchronization. Participants tapped a familiar melody at a comfortable and regular rate to assess their SPRs. They then synchronized their tapping of the same familiar melody with a metronome set to their SPRs as well as to rates proportionally faster and slower than their SPRs. We predicted that non-musicians would show faster SPRs than musicians, consistent with previous research on SMT (Drake et al., 2000a). We applied linear (lag-1 autocorrelation) and non-linear (recurrence quantification analysis) time series analyses to investigate rate flexibility as a function of temporal error correction as well as dynamic patterns of behavior across longer timescales. Finally, individuals’ hand measurements were collected to further explore potential contributions of peripheral mechanisms to SPRs. If spontaneous rates represent attractor states within the space of possible production rates, then biases in synchronization accuracy should center around an individual’s spontaneous rate. We hypothesize that this will be shown by lagging at faster rates and anticipating at slower rates relative to the spontaneous rate. In addition, we hypothesize that spontaneous rates should act as a weaker attractor for musicians than for non-musicians, resulting in greater synchronization accuracy for musicians across rates.

## Experiment 1

### Methods

#### Participants

Twenty pianists (mean years old = 21, *SD* = 3; 14 females) with at least 6 years of private piano instruction (mean years private instruction = 12 years; *SD* = 3) were recruited to participate in this study. Only right-handed pianists were included. Handedness was confirmed using the Edinburgh Handedness Inventory (Oldfield, 1971). All participants were right-handed (16 pianists) or had indeterminate handedness with a tendency toward right-handedness (4 pianists), as determined by their scores (ranging from 33.33 to 100). All participants exhibited normal hearing in the frequency range of stimuli used in the experiment (<30 dB HL threshold for 125–750 Hz), as determined by an audiometry screening. Although there were no neurological or speech disorder exclusion criteria, we report the neurological histories here of the participants: Two participants reported a history of concussions, and one participant reported a lisp. These participants met all of the inclusion criteria and their data yielded the same patterns of results as the other participants.

#### Equipment and Stimuli

Hearing screenings were administered with a Maico MA 40 audiometer. Hand measurements were taken from tracings of participants’ right (dominant) hands with a 12-inch Capri digital caliper. Participants performed and tapped melodies on a Yamaha PSR-500M electronic keyboard. Auditory feedback from the keyboard was delivered to participants in a piano timbre via a Roland Studio Canvas SD-50 through Bose QC 20 noise-canceling headphones. FTAP (Finney, 2001) was used to generate auditory feedback and record MIDI tap timing on a computer (Dell T3600) running Linux (Fedora 16).

Stimulus melodies were chosen for their familiarity among participants and their simple rhythms. Practice melodies were chosen for the purpose of teaching participants the novel task, and consisted of “London Bridge is Falling Down,” “If You’re Happy and You Know It Clap Your Hands,” and “Happy Birthday to You,” all composed in D Major. Experimental melodies used in the actual trials after participants were comfortable with the novel task consisted of “Twinkle, Twinkle Little Star,” composed in G Major (Stimulus 1), and “Mary Had a Little Lamb,” composed in F Major (Stimulus 2). Each melody consisted of a 8–12 measure tune of binary (4/4) meter, performed by the right hand, which was notated with suggested fingerings.

Questionnaires included the Edinburgh Handedness Inventory (Oldfield, 1971), which assessed the degree to which an individual was right- or left-handed, and a musical background questionnaire that assessed participants’ age, level of education, musical training background, musical experience (i.e., listening and performance), as well as any hearing, speech, or neurological problems.

#### Design

The experiment used a within-subjects design with two independent variables of Melody (Stimulus 1 and Stimulus 2) and Task (Piano Performance and Tapping). The dependent variable was the SPR (see section “Data Analysis”). Participants always completed the Piano Performance task first to ensure that they learned the correct rhythm of each melody for the Tapping task. Melodies were blocked within task; melody order within task was randomized for each participant, and this order was held constant across tasks.

#### Procedure

All participants gave informed consent upon arrival at the lab. In the first part of the experiment, participants completed an audiometry screening. Participants were then given the names of the practice and experimental melodies to assess their familiarity with these melodies. Participants who passed the audiometry screening and were familiar with at least one practice melody and both experimental melodies upon hearing only the melody names were eligible to participate. Tracings were then taken of each participant’s right (dominant) hand, marking the first crease of the wrist. Hand measurements were taken from these tracings from the radius to the ulna at the wrist (wrist width), from the first crease of the wrist to the tip of the third digit (middle finger), and from the ulna at the wrist to the tip of the first digit (thumb).

Next, participants were given music notation for the first experimental melody and were asked to practice the melody until it was memorized. Participants were instructed to write their chosen fingering on the notation if it differed from the suggested fingering. Once the melody was memorized, the notation was removed and participants completed one practice trial in which they played the melody four times through without stopping to ensure that the melody was memorized without pitch errors. Following the practice trial, participants completed three test trials in which they performed the same melody four times through without stopping between melody repetitions at a comfortable and steady rate. This procedure was then repeated for the second experimental melody. Both practice and test trials were checked for pitch errors by computer comparison with the notated score (described in section “Data Analysis”) (Large, 1993; Rankin et al., 2009).

Participants next completed the musical background questionnaire and the Edinburgh Handedness Inventory (Oldfield, 1971), lasting approximately 5–10 min, which created a short break between the piano performance and tapping tasks to reduce the likelihood that consistency in SPRs across tasks could be explained by recent exposure to the piano performance rate. During this break, a blanket was placed over the entire keyboard except for the ends of the white keys for the tapping task to reduce associations with music performance that might be primed by visual exposure to the keyboard.

Participants next performed the tapping task in which they were instructed to tap the rhythm of the familiar melody on a single key of the keyboard with the index finger of their dominant (right) hand. They were told that each time they tapped, a melody tone would sound. Participants first completed a practice trial with one of the practice melodies, during which they tapped the melody four times continuously. Once participants felt comfortable with the tapping task, they moved on to the experimental trials. Participants completed a practice trial with the first experimental melody. Then participants completed three test trials in which they tapped the melody four times without stopping between melody repetitions at a comfortable and steady rate. This procedure was then repeated for the second experimental melody.

#### Data Analysis

Participants’ SPRs were computed as the mean inter-onset interval (IOI) across the middle two repetitions of the melody in each test trial to capture participants’ maximally stable behavior (Loehr and Palmer, 2011; Zamm et al., 2015, 2016). In piano performances, pitch errors were identified by computer comparison of the performance with the pitch contents based on the music notation for that melody using the MIDI Matcher program in Matlab (Large, 1993; Rankin et al., 2009). Melody repetitions that contained pitch errors (added or deleted tones) were excluded from analysis (2.1% of all repetitions). Tapping trials did not contain pitch errors because pitches were produced by a computer. Half notes in the melodies were interpolated to extract the quarter note pulse for calculation of the mean IOI and the final (whole) note in each melody repetition was excluded to avoid bias in quarter note pulse estimation. Outlier IOIs defined as values more than three standard deviations away from the mean were excluded from analysis (piano performance trials = 1.0% of all IOIs; tapping trials = 1.1% of all IOIs).

### Results

**FIGURE 1:**
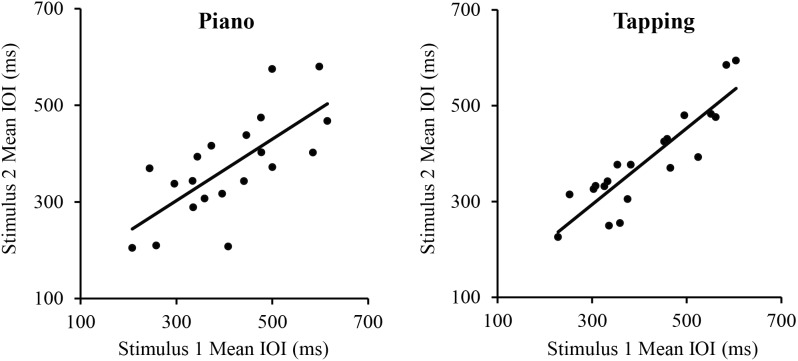
Correlations between pianists’ Spontaneous Rate values (mean IOIs) for stimulus melodies 1 and 2. Left panel: piano performance task; Right panel: tapping task. Each point represents a single participant.

**FIGURE 2:**
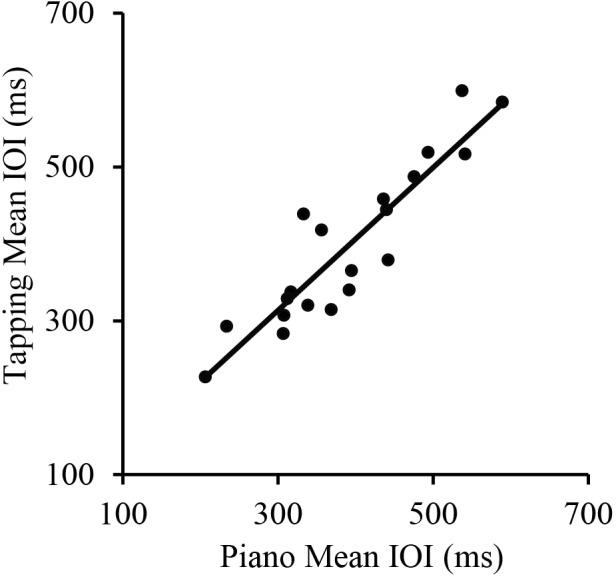
Correlations between pianists’ Spontaneous Rate values (mean IOI across melodies) for the piano performance task and tapping task. Each point represents a single participant.

We first assessed the consistency of SPRs across the two Melodies within each Task. Simple correlations showed that SPRs were consistent across Melodies for both the Piano Performance Task, *r*(18) = 0.71, *p* < 0.001 and the Tapping Task, *r*(18) = 0.89, *p* < 0.001, shown in **Figure 1**. SPRs were then collapsed across Melodies within Tasks for each individual to compare each individual’s Piano Performance SPR with their Tapping SPR. The simple correlation of individual SPRs from Piano Performance and Tapping Tasks, shown in **Figure 2**, yielded highly consistent SPRs across Tasks, *r*(18) = 0.91, *p* < 0.001.

Next, we tested whether SPRs differed across Tasks and Trials. A two-way analysis of variance (ANOVA) conducted on the mean IOI of each performance by Task (Piano Performance and Tapping) and Trial (1, 2, and 3) showed that there was no main effect of Task, *F*(1,19) = 0.52, *p* = 0.48, indicating that the mean SPRs for Piano Performance (mean = 391 ms) and Tapping (mean = 398 ms) did not differ significantly. A main effect of Trial, *F*(2,38) = 19.94, *p* < 0.001 indicated small but slightly faster rates across Trials (Trial 1 mean = 402 ms; Trial 2 mean = 394 ms; Trial 3 mean = 388 ms; Tukey’s HSD = 5.21, *p* < 0.05). There was no interaction between Task and Trial, *F*(2,38) = 1.39, *p* = 0.26.

We next investigated the relationship between SPRs and hand measurements to test whether hand size contributed to individual differences. Piano Performance SPR did not correlate significantly with any of the hand measurements (Wrist to Digit 3: *r*(18) = 0.02, *p* = 0.93; Wrist Width: *r*(18) = 0.31, *p* = 0.18; Wrist to Digit 1: *r*(18) = 0.13, *p* = 0.59). The relationship between Tapping SPR and the Wrist Width measurement was marginally significant following Bonferroni correction for multiple comparisons, *r*(18) = 0.49, *p* = 0.09. There were no other correlations between Tapping SPR and hand measurements (Wrist to Digit 3: *r*(18) = 0.16, *p* = 0.51; Wrist to Digit 1: *r*(18) = 0.28, *p* = 0.23).

**FIGURE 3:**
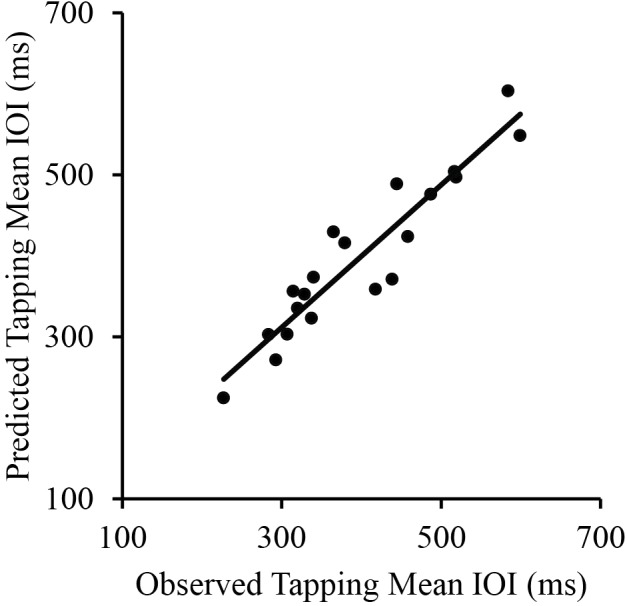
Correlations between pianists’ observed tapping mean IOI and their predicted tapping IOI based on their piano performance SPR (mean IOI) and wrist width. Each point represents a single participant.

To further investigate the independent contributions of Piano Performance SPR and Wrist Width to Tapping SPR, a multiple regression was conducted to predict Tapping SPR from Piano Performance SPR and Wrist Width. Piano Performance SPR and Wrist Width provided a significant fit as predictors of Tapping SPR (*R* = 0.94, *p* < 0.001; **Figure 3**). Together, the variables accounted for 88% of the variance. Semi-partial correlation coefficients were significant both for Piano Performance SPR, β = 0.84, *t*(17) = 9.50, *p* < 0.001, and Wrist Width, β = 0.23, *t*(17) = 2.55, *p* < 0.05, indicating significant contributions of each variable to Tapping SPR.

### Discussion

Experiment 1 demonstrated the consistency of spontaneous rates across piano performance and tapping, two tasks that differ in their motor complexity due to differences in the number of fingers used to execute each task (the tapping task required single-finger movement; the piano performance task required 5-finger movement) and the number of spatial dimensions of movement (the tapping task required up/down movement; the piano performance task required lateral (left/right) movement in addition to up/down movement). The consistency of SPRs across tasks provides validation of the tapping task as eliciting a rate-specific experience similar to piano performance. One benefit of the novel task is that it can be used with any participant who needs only to be familiar with the rhythm of a melody to be able to produce the melody. Another benefit is that the task is equally novel to musicians and non-musicians.

These findings also provide further support for the SPR as reflecting a preferred coordination mode at which movement is optimized. Biomechanical views that predict movement rates as reflecting task-specific motor demands (Goodman et al., 2000) are not consistent with the similarities we observed in SPRs for 5-finger lateral movements typical of piano performance and 1-finger vertical movements typical of tapping. Instead, these results suggest at least some contribution to these rates from central timing mechanisms (Latash, 1992).

We also found significant contributions of both wrist width and piano performance SPR to tapping SPR, consistent with previous research showing that wrist movements contribute most to freestyle single-finger tapping (Dennerlein et al., 2007). Wrist width is an anatomical (rather than kinematic) measurement; specifically, wrist width has been shown to provide an accurate measure of frame size (Himes and Bouchard, 1985). Wrist width as a predictor of tapping SPR may therefore reflect the additional influence of body frame (i.e., larger body frame, slower tapping SPR) in the tapping task. We pursue this finding further in Experiment 2 to better understand contributions of wrist size to SPRs.

## Experiment 2

Experiment 2 implemented the novel musical tapping task with musicians and non-musicians to examine the effects of spontaneous rates and musical training on rate flexibility of synchronization. While previous research has independently shown that spontaneous rates constrain synchronization (Loehr and Palmer, 2011; Palmer et al., 2013; Zamm et al., 2015, 2016) whereas musical training enhances it (Drake et al., 2000a,b; Aschersleben, 2002), little is known about how musical training influences the flexibility with which individuals can move away from their spontaneous rates. Musicians and non-musicians tapped a familiar melody at a comfortable and regular rate as a measure of their SPRs. They then synchronized their tapping of the same melody with a metronome cue calibrated to a range of rates centered around each individual’s SPR. Additionally, participants’ wrist width measurements were taken to further examine the relationship between wrist width and tapping SPR.

### Methods

#### Participants

Twenty musicians (mean years old = 21; *SD* = 2; 17 females) and 20 non-musicians (mean years old = 23; *SD* = 4; 17 females) participated in the experiment. None of the musicians had participated in the previous experiment. Musicians had at least 6 years of private instrumental music instruction (mean years private instruction = 10; *SD* = 3). Percussionists were excluded because they have been shown to have superior synchronization abilities compared with other musicians (Krause et al., 2010). Non-musicians had no private music instruction in the past 6 years, and had less than 2 years of private music instruction overall (mean years private instruction = 0.4; *SD* = 0.6). Most musicians (18) and non-musicians (19) were right-handed; one musician had indeterminate handedness with a tendency toward right-handedness, one non-musician had indeterminate handedness with a tendency toward left-handedness, and one musician was left-handed as determined by their scores (ranging from -50 to 100) on the Edinburgh Handedness Inventory (Oldfield, 1971). All participants had normal hearing in the frequency range of stimuli used in the experiment (<30 dB HL threshold for 125–750 Hz), as determined by an audiometry screening, and stated being familiar with the melodies used in the experiment. Additionally, all participants had normal pitch perception as determined by the Montreal Battery of Evaluation of Amusia (MBEA) scale subtest (Peretz et al., 2003). Although there were no neurological or speech disorder exclusion criteria, we report the neurological histories here of the participants: One participant reported a history of concussions, three participants reported a history of epilepsy (one in childhood), and one participant reported a lisp. These participants met all of the inclusion criteria and their data yielded the same patterns as the other participants. Participants included in the experiment showed successful synchronization in at least one trial in all rate conditions, as determined by the Rayleigh test (see section “Data Analysis”); 8 additional non-musicians were excluded due to failure to synchronize in one or more rate conditions. Groups did not differ significantly in age (musicians = 20.80; non-musicians = 22.50; *t*(1) = 3.41, *p* = 0.07) or years of education (musicians = 14.93; non-musicians = 16.13; *t*(1) = 2.81, *p* = 0.10).

#### Equipment and Stimuli

Participants’ hearing thresholds were tested with a Maico MA 40 audiometer. Hand measurements were taken with a 12-inch Capri digital caliper. Participants tapped the rhythms of melodies on a force-sensitive resistor (FSR) of an Arduino connected via a MIDI cable to a computer (Dell T3600) running FTAP (Finney, 2001) on Linux (Fedora 16). Based on timing tests conducted with a Tektronix TDS 2002 oscilloscope, the time from the start of the tap on the FSR to the start of the MIDI signal sent from the Arduino averaged 1.0 millisecond (*SD* = 0.035; see Supplementary Figures 1, 2). Auditory feedback consisted of a familiar melody in a piano timbre and a metronome in a woodblock timbre, delivered to participants via a Roland mobile studio canvas SD-50 through AKG headphones at a comfortable listening level.

Stimuli consisted of a subset of the melodies from Experiment 1: “Happy Birthday to You” and “Mary Had a Little Lamb.” These melodies were chosen for their familiarity among participants from the previous experiment as well as for their simple rhythms. “Happy Birthday to You,” composed in D Major, was used as a practice melody to teach participants the tapping task. “Mary Had a Little Lamb,” composed in F Major, was used as the experimental melody.

#### Design

The experiment consisted of three main tasks: a SPR task, a rate flexibility task, and a maximal rate task, performed in that order. During the SPR task, participants tapped the experimental melody at a comfortable and steady rate. Performances from this task were used to calculate the SPR (see section “Data Analysis”). In the rate flexibility task, participants synchronized their tapping of the experimental melody with a metronome at five different rates: 30% slower than the SPR, 15% slower than the SPR, the SPR, 15% faster than the SPR, and 30% faster than the SPR. The rate flexibility task had a mixed design with a between-subject variable of Group (Musician and Non-musician) and within-subject variable of Metronome Rate (30% Slower, 15% Slower, the SPR, 15% Faster, and 30% Faster). The order of rates was pseudo-randomized such that participants never received rates in order from either slowest to fastest or fastest to slowest. Finally, participants completed the maximal rate task in which they tapped the experimental melody as fast as possible while maintaining the rhythm of the melody; the goal of this task was to assess participants’ motor limits.

#### Procedure

All participants gave informed consent upon arrival at the lab. First, participants completed an audiometry screening and a familiar melody assessment to confirm normal hearing and familiarity with the stimulus melodies. Participants who did not pass these assessments were excluded from the experiment. Next, the hand measurement from the radius to the ulna at the wrist (wrist width) was taken of each participant’s dominant hand using a digital caliper.

Participants were next taught how to perform the tapping task using the familiar practice melody. Participants were seated next to a table on which the Arduino was placed at a comfortable height such that participants could rest their arm while tapping. Participants were instructed to tap the rhythm of the melody with the index finger of their dominant hand in the middle of the FSR, and that each time they tapped, a tone of the melody would sound. Participants practiced the task with a practice melody until they were comfortable with the task.

Participants then completed the SPR task with the experimental melody. Participants first completed a practice trial in which they tapped the melody at a comfortable and steady rate. Participants were instructed that they should continue tapping the melody four times through without stopping between melody repetitions until they no longer heard the melody tones, a signal that the trial was over. After completing one practice trial, the experimenter gave participants extra practice as was necessary and participants completed three experimental trials. At the end of the SPR task, participants’ SPRs were calculated (see section “Data Analysis”) to determine the rate conditions for the rate flexibility task. During this time, participants completed a musical background questionnaire and the Edinburgh Handedness Inventory (Oldfield, 1971).

At the beginning of the rate flexibility task, participants were instructed that they would hear a metronome, and that they should start synchronizing their tapping of the same experimental melody with the metronome within the first eight metronome clicks. Participants were instructed to synchronize each tap with a metronome click and to continue synchronizing their tapping of the melody with the metronome for four melody repetitions without stopping between repetitions until they no longer heard the melody tones, which would signal the end of a trial. Participants first completed a practice trial in which they synchronized with the metronome at one of the 5 metronome rates. The experimenter gave participants extra practice as was necessary. Next, participants completed three test trials. Extra trials were given if participants made mistakes, such as not tapping on the FSR. This procedure was repeated for the remaining rate conditions.

Participants then completed the maximal rate task. Participants were instructed that they would tap the same experimental melody as fast as possible (without a metronome) while maintaining the rhythm of the melody. Participants were instructed to tap the melody only once through. Participants completed one practice trial and one test trial.

Finally, participants completed the scale subtest from the MBEA (Peretz et al., 2003). In each trial, two short melodies were presented over headphones and participants indicated whether the two melodies were the same or different in pitch.

#### Data Analysis

Participants’ SPRs were calculated from the SPR task as before: the mean IOI across the middle two repetitions of each trial. Outlier IOIs more than three standard deviations from the mean were excluded (% of total IOIs per group: musicians = 0.8%; non-musicians = 1%). Participants’ maximal rates were calculated in the same way; calculations were based on only one repetition because participants only completed one test trial in which they tapped the melody once through.

Synchronization trials were analyzed by aligning taps and metronome clicks in each trial using a nearest neighbor approach, similar to Pecenka and Keller (2011). The signed asynchronies were calculated as the tap minus metronome onset, such that a negative value indicates that a tap preceded the metronome and a positive value that a tap lagged the metronome. For each participant, asynchrony outliers more than 3 standard deviations from their mean were removed from analysis (% of total asynchronies per group: musicians = 0.6%; non-musicians = 0.8%). Taps that did not align with a metronome onset were discarded, as participants were instructed to synchronize each tap with a metronome click. Given that the non-musician group showed a wide range of synchronization abilities, the Rayleigh test for circular non-uniformity was first implemented to determine trials containing a unimodal synchronization pattern (Fisher, 1993). This test is implemented by computing relative phase (asynchrony divided by the metronome IOI), and then converting to degrees. This test is sensitive to unimodal departures from uniformity, with a significant result indicating a unimodal distribution. Following previous synchronization measures (Kirschner and Tomasello, 2009; Pecenka and Keller, 2011; Sowiński and Dalla Bella, 2013; Dalla Bella and Sowiński, 2015), trials in which the null hypothesis of circular uniformity could not be rejected (i.e., the distribution was not unimodal) at *p* < 0.05 were excluded from further analysis (musicians: 0% of trials excluded; non-musicians: 9% of trials excluded).

Finally, adjusted synchronization accuracy measures for each individual were defined as mean asynchrony in each individual’s rate condition minus the mean asynchrony in that individual’s SPR rate condition. The adjusted synchronization measures allowed us to examine the pattern of synchronization relative to the SPR, and to compare synchronization accuracy across musician and non-musician participants with different baseline synchronization abilities.

### Results

#### Spontaneous Production Rates

**FIGURE 4:**
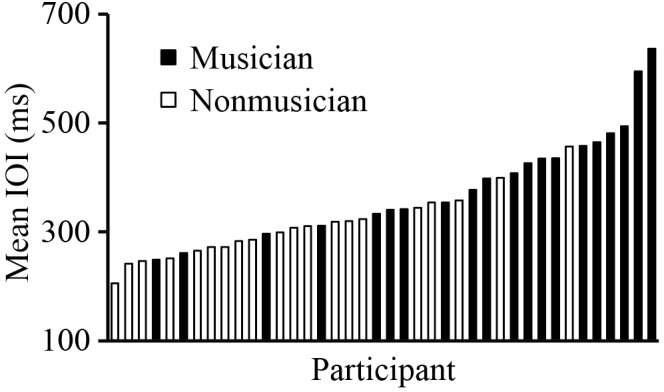
Distribution of SPR values (mean IOI) across participants. Each bar represents the SPR of a single participant; black bars are musicians (group mean = 405 ms) and white bars are non-musicians (group mean = 306 ms).

We first investigated whether SPRs differed between Musicians and Non-musicians, and whether SPRs were stable across Trials. **Figure 4** shows the distribution of SPRs. A mixed analysis of variance (ANOVA) by Group (Musician and Non-musician) and Trial (1, 2, and 3) showed a significant main effect of Group, *F*(1,38) = 14.48, *p* < 0.01; Musicians’ SPRs (mean = 405 ms) were slower on average than Non-musicians’ SPRs (mean = 306 ms). There was also a significant main effect of Trial, *F*(2,76) = 13.71, *p* < 0.001. *Post hoc* comparisons revealed that Trial 1 (mean = 363 ms) was slightly slower than Trials 2 and 3 (mean Trial 2 = 353 ms; Trial 3 = 350 ms) (Tukey’s HSD = 5.84, *p* < 0.05), likely reflecting an adjustment to the task during Trial 1. There was no significant interaction between Group and Trial, *F*(2,76) = 1.15, *p* = 0.32.

We also investigated the stability of the SPR, measured by the coefficient of variation (CV; standard deviation of the IOIs divided by the mean IOI). The same ANOVA repeated on the CVs in the SPR task showed a significant main effect of Group, *F*(1,38) = 11.08, *p* < 0.01; Musicians were less variable at their SPR (mean = 0.05) than Non-musicians (mean = 0.06). There was no main effect of Trial, *F*(2,76) = 0.77, *p* = 0.47, or interaction between Group and Trial, *F*(2,76) = 0.50, *p* = 0.61, suggesting that SPRs were stable across time for all participants.

#### Synchronization Accuracy

**FIGURE 5:**
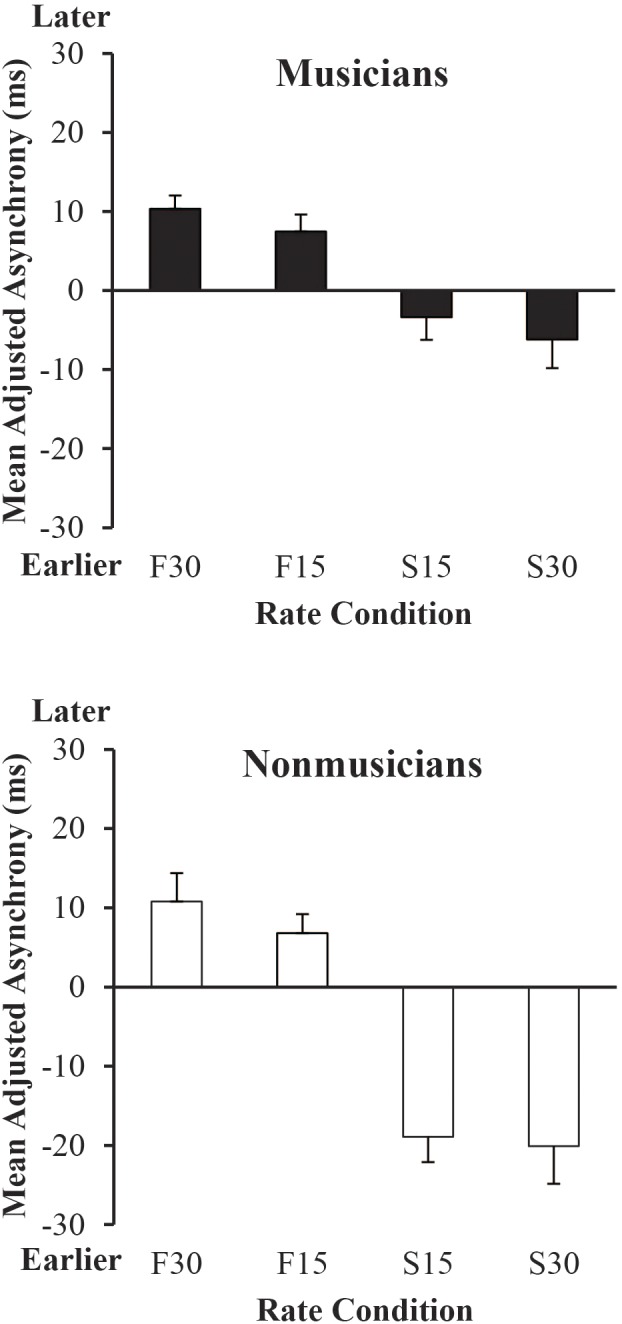
Mean adjusted signed asynchronies (each participant’s rate condition mean asynchrony minus SPR condition mean asynchrony) by rate condition and group. Error bars show standard error. Positive values (earlier) indicate asynchrony values for which the participant tapped sooner and negative values indicate asynchrony values for which the participant tapped later relative to their performance in the SPR condition.

Next, we examined the adjusted synchronization accuracy by Group and Metronome Rate, shown in **Figure 5**. A mixed ANOVA indicated a significant main effect of Group, *F*(1,38) = 6.99, *p* < 0.05, a significant main effect of Metronome Rate, *F*(3,114) = 37.01, *p* < 0.001, and a significant interaction between Group and Metronome Rate, *F*(3,114) = 4.43, *p* < 0.01. *Post hoc* comparisons revealed that Non-musicians anticipated more at the 30% Slower than at 15% and 30% Faster rates, and at the 15% Slower than at 15% and 30% Faster rates (Tukey’s HSD = 12.41, *p* < 0.05). Musicians anticipated more at the 30% Slower than at 15% and 30% Faster rates, and at the 15% Slower than 30% Faster rate. Finally, Musicians synchronized more accurately than Non-musicians at both 15% and 30% Slower rates.

We also examined which Metronome Rate conditions in **Figure 5** differed from 0, where 0 is participants’ synchronization accuracy at the SPR. *T*-tests (Bonferroni-adjusted) of mean values at each Metronome Rate against 0 indicated that Musicians lagged significantly at 15% and 30% Faster rates relative to 0 (*p*’s < 0.01). Non-musicians lagged significantly at 15% (*p* = 0.01) and 30% (*p* < 0.01) Faster rates and also anticipated significantly at 15% and 30% Slower rates (*p*’s < 0.01) relative to 0.

To compare Musicians’ and Non-musicians’ synchronization accuracy across rates, the slope of each individual’s adjusted mean signed asynchronies was computed by predicting the adjusted mean signed asynchronies shown in **Figure 5** from the prescribed metronome IOI (ms) for each rate condition. A one-way ANOVA on each participant’s slope values from these regression fits indicated a significant main effect of Group, *F*(1,38) = 8.90, *p* < 0.01. Musicians had shallower slopes (mean = -0.08) than Non-musicians (mean = -0.19), indicating greater synchronization accuracy across rates.

#### Maximal Rates

To ensure that observed effects of Group and Metronome Rate on synchronization were not driven by biomechanical limits at participants’ fastest metronome rates, maximal rates were compared across Groups and were also compared with participants’ fastest metronome rate (30% Faster rate). A one-way ANOVA on maximal rates showed no significant main effect of Group, *F*(1,38) = 0.37, *p* = 0.55 (Musicians’ mean maximal rate = 172 ms; Non-musicians’ mean maximal rate = 164 ms). A one-way ANOVA on CV of maximal rates also showed no significant main effect of Group, *F*(1,38) = 1.79, *p* = 0.19 (Musicians’ mean CV = 0.06; Non-musicians’ mean CV = 0.07).

Participants’ maximal rates were then compared with the fastest synchronization condition (30% Faster) to ensure that participants did not reach ceiling on their possible movement rates. Thirty-eight of the 40 participants showed maximal tapping rates faster than their 30% Faster prescribed (metronome-determined) rate. For the two exceptions (both Non-musicians), participants’ maximal rates were then compared with their 30% Faster observed (tapping) rate to ensure that their maximal rates reflected their fastest tapping rate. These comparisons showed that both participants had faster 30% Faster observed rates than maximal rates. These findings suggest that no participant reached ceiling on their possible movement rates during the rate flexibility task.

#### Lag-1 Autocorrelation

**FIGURE 6:**
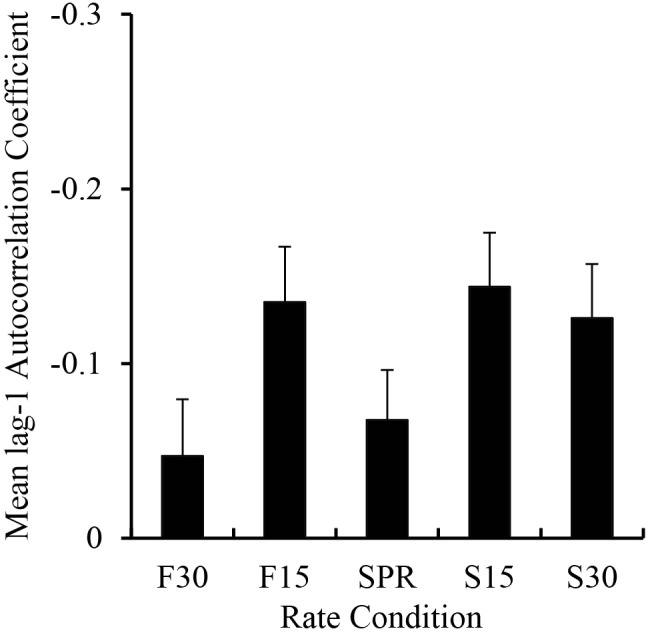
Mean lag-1 autocorrelation coefficients computed on the tapping IOI sequences by rate condition. Negative values are plotted upward. Error bars show standard error.

To investigate Musicians’ enhanced synchronization across the range of rates, we ran a lag-1 autocorrelation on the IOIs in each rate condition to test the potential role of error correction (Wing and Kristofferson, 1973). Similar to error correction analyses of poor synchronizers (Sowiński and Dalla Bella, 2013), the analysis included the first melody repetition in each trial with the fewest IOI outliers. A mixed ANOVA on the lag-1 autocorrelation coefficients per trial by Group and Metronome Rate showed a significant main effect of Group, *F*(1,38) = 11.16, *p* < 0.01, such that Musicians showed more negative lag-1 autocorrelations (mean = -0.18) than Non-musicians (mean = -0.03). Results also showed a significant main effect of Metronome Rate, *F*(4,152) = 5.19, *p* < 0.01; the lag-1 autocorrelation coefficients are shown by Metronome Rate in **Figure 6**. *Post hoc* comparisons revealed larger negative values in the 30% Slower than 30% Faster rate, 15% Slower than SPR and 30% Faster rates, and 15% Faster than 30% Faster rate. There was no interaction between Group and Metronome Rate, *F*(4,152) = 1.03, *p* = 0.39. Thus, Musicians showed more error correction than Non-musicians and both Groups showed more error correction at slower rates.

#### Recurrence Quantification Analyses

To further investigate differentiating characteristics of coordination between Musicians and Non-musicians across a longer timescale, we ran autorecurrence quantification analyses (RQA) on the asynchrony time series. Whereas the lag-1 autocorrelation investigates error correction at a local timescale (Sowiński and Dalla Bella, 2013), RQA measures provide information about recurring behavioral patterns across longer timescales (Schmit et al., 2005, 2006; Richardson et al., 2008). Because we were interested in comparing optimal performances of Musicians and Non-musicians, RQA was applied to the SPR Metronome Rate condition, a conservative test between Groups because the SPR condition was tailored to be most comfortable for all participants. If Non-musicians show RQA patterns that indicate temporal rigidity in synchronization, this could provide a possible mechanism for their reduced flexibility in synchronizing with other rates. The RQA analysis was applied to all data (including outliers) to preserve the time series, and to asynchronies (rather than IOIs) to allow that Non-musicians did not always tap the melody rhythm as expected, and thus the asynchrony measures provided longer uninterrupted time series for analysis.

Consistent with previous RQA implementations, (Schmit et al., 2005, 2006; Richardson et al., 2008), we examined the outcome measures of percent recurrence (how often a data point is repeated in a time series, related to regularity), percent determinism (how often recurrent points form lines, an inverse measure of randomness), and maxline (measure of the response of a system to changes in initial conditions, also known as mathematical stability). Based on tests of a wider range of parameter values that generated similar patterns of results, final parameter settings for the analysis were: time delay = 14 samples (which coincided with approximately half of the stimulus melody cycle), embedding dimensions = 7, radius = 60% of the mean distance between data points in the reconstructed phase space. The number of successive points required to form a line was set to 2. These parameters were selected to obtain percent recurrence of at least 1% for each trial without reaching a ceiling of 100% determinism (Schmit et al., 2005).

**FIGURE 7:**
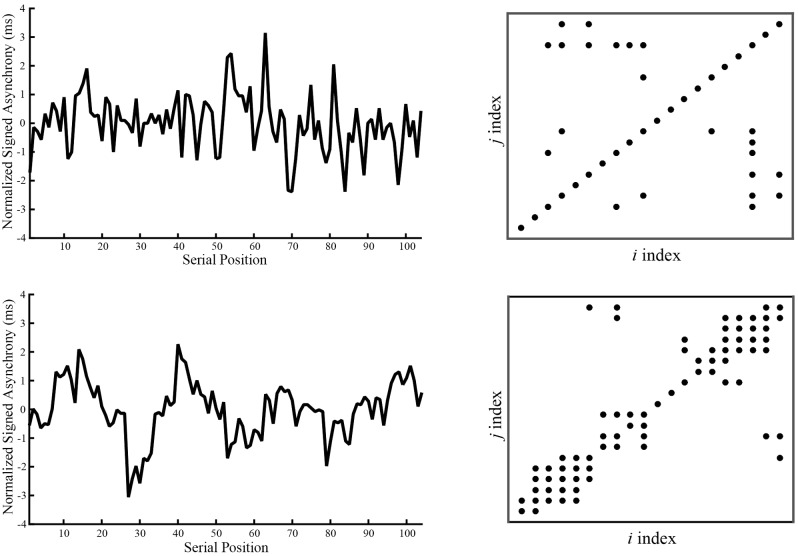
Sample normalized signed asynchronies (left graphs) and recurrence plots (right) for a single trial for one musician (top row) and one non-musician (bottom row).

The mean values for percent recurrence, percent determinism, and maxline per Group are shown in **Table 1**. ANOVAs conducted by Group for each measure showed a main effect of Group on percent recurrence, *F*(1,38) = 15.70, *p* < 0.001, percent determinism, *F*(1,38) = 24.94, *p* < 0.001, and maxline, *F*(1,38) = 31.05, *p* < 0.001. Non-musicians had significantly higher recurrence (Non-musicians = 6.83%; Musicians = 4.48%), higher determinism (Non-musicians = 36.03%; Musicians = 12.99%), and higher maxline (Non-musicians = 2.18; Musicians = 0.73) than Musicians. Thus, Non-musicians showed more regularly patterned behavior than Musicians. Exemplar plots for a single trial by a Musician and a Non-musician are shown in **Figure 7**.

**Table 1:** Group comparisons of RQA outcomes.

RQA Measure	Musicians	Non-musicians
% Recurrence	4.48%	6.83%
% Determinism	12.99%	36.03%
Maxline	0.73	2.18


**p*’s < 0.001.*

#### Individual Differences in Rate Flexibility

We examined the relationship between each performer’s rate flexibility in synchronization (represented by the slope value for the asynchronies across rates in **Figure 5**), and the measures of temporal variability in spontaneous production (CV), feedback correction (mean lag-1 autocorrelation of IOIs across rates), and recurrence patterns during synchronization at the SPR (% determinism). These measures were chosen because they showed group differences between Musicians and Non-musicians. Multiple regressions were conducted separately for each group with CV, lag-1 autocorrelation, and % determinism as predictors for asynchrony slope (larger asynchronies across rates were represented by a more negative slope value); two participants were excluded due to outlier values for the measures of CV (1 Non-musician) and lag-1 autocorrelation (1 Non-musician). The multiple regression analysis indicated a significant fit for Musicians (*R* = 0.79, *p* < 0.001). Semi-partial correlation coefficients indicated that only the CV values predicted the asynchrony slope values above and beyond other variables, β = -0.73, *t*(16) = 3.41, *p* < 0.01 (lag-1 autocorrelation, β = -0.09, *p* = 0.64; % determinism, β = -0.03, *p* = 0.87). In contrast, the multiple regression model did not provide a significant fit for Non-musicians’ slope values (*R* = 0.21, *p* = 0.88). Thus, individual differences in Musicians’ temporal variability in unpaced (spontaneous) performance was predictive of rate flexibility; the more regular their unpaced performance (CV closer to 0), the closer to 0 (less negative) their slope value.

#### Wrist Width

Finally, we examined the relationship between participants’ wrist width measures and their SPRs. An ANOVA on wrist width measures by Group showed no significant differences, *F*(1,38) = 0.56, *p* = 0.46. Next, a correlation conducted across Groups indicated that wrist width was not significantly correlated with the SPR, *r*(38) = -0.18, *p* = 0.26. As well, wrist width was not significantly correlated with the SPR within either Group: for Musicians, *r*(18) = -0.26, *p* = 0.27, or Non-musicians, *r*(18) = 0.04, *p* = 0.88. Nor was wrist width correlated with the slope values from participants’ adjusted mean asynchronies across rates (*r* = 0.03, *p* = 0.87). These findings suggest that biomechanical factors alone did not account for Group differences in SPR.

### Discussion

The second experiment implemented the novel musical tapping task validated in the first experiment with musicians and non-musicians to investigate the effects of musical training and spontaneous rates on rate flexibility of synchronization. Participants’ SPRs were measured as they tapped the rhythm of a familiar melody at a comfortable and steady rate. Participants then synchronized their tapping of the melody with a metronome set at their SPRs and rates proportionally slower and faster than their SPRs.

First, musicians’ SPRs were significantly slower than non-musicians’ SPRs. This finding is consistent with previous research on SMT in which musicians show slower tapping rates than non-musicians (Drake et al., 2000a). Additionally, musicians were less variable than non-musicians during self-paced performances at their SPRs. Given that the SPR task required participants to produce the rhythm in accordance with the corresponding melody tones, non-musicians’ increased variability in this task may arise from weaker auditory-motor integration (Pfordresher and Brown, 2007; Sowiński and Dalla Bella, 2013).

Second, both musicians and non-musicians anticipated more at slower rates and lagged more at faster rates relative to their SPRs in the synchronization task. This finding indicates a constraint placed on rate flexibility by the SPR for all participants, consistent with results from studies on duet synchronization (Loehr and Palmer, 2011; Zamm et al., 2015, 2016). Interestingly, non-musicians synchronized less accurately than musicians at slower rates, whereas both groups performed similarly at faster rates. This finding, together with non-musicians’ faster SPRs, suggests that non-musicians may have a bias toward faster rates. This bias may indicate that non-musicians are more restricted in their ability to track events over longer timescales compared with musicians (Drake et al., 2000b). Importantly, musicians synchronized more flexibly across rates than non-musicians, as indicated by their similar synchronization accuracy between rates close to their SPRs (i.e., 15% slower and faster rates) and by their smaller slope values (less change across rates) of the adjusted asynchronies. Additionally, the temporal regularity of unpaced performance significantly predicted synchronization performance across rates for musicians but not for non-musicians, suggesting different mechanisms driving rate flexibility across groups.

Third, musicians demonstrated larger negative lag-1 autocorrelations of IOIs compared with non-musicians. These findings suggest that musicians engage in more error correction than non-musicians, which may contribute to their enhanced synchronization accuracy across rates (Sowiński and Dalla Bella, 2013). Additionally, RQA indicated that non-musicians had higher recurrence (repetition), determinism (lower randomness), and maxline (mathematical stability) than musicians, suggesting more rigidity in their synchronization behaviors than musicians.

## General Discussion

The current experiments investigated the roles of spontaneous rates and musical training on rate flexibility of synchronization. Experiment 1 introduced a novel musical tapping task appropriate for measuring spontaneous rates in both musicians and non-musicians; that experiment showed that pianists produced familiar melodies at highly consistent rates across 5-finger piano performance and 1-finger tapping. Experiment 2 implemented the novel task to show that musicians synchronized more flexibly across rates than non-musicians, and group differences in rate flexibility could be characterized both by differences in error correction and in dynamic patterns of synchronization over time.

Notably, all participants showed some constraint of SPRs on synchronization accuracy at other rates, indicated by the tendency to anticipate more at slower rates and lag more at faster rates relative to each individual’s SPR. Musicians’ temporal stability of performances at the SPR were correlated with their synchronization flexibility across rates: the less variable musicians were in unpaced performance, the more synchronous their paced performances were across rates. This finding of increased temporal stability for performances at the SPR as an indicator of synchronization flexibility across rates is consistent with the interpretation of the spontaneous rate as a natural frequency at which minimum energy expenditure is required to coordinate movement across the parts of a system (Von Holst, 1937; Haken et al., 1985; Kelso, 1997), such as finger and wrist joints in tapping.

Musicians and non-musicians differed on several performance measures. First, musicians synchronized more accurately across rates than non-musicians, suggesting that rate flexibility is enhanced by musical training. Furthermore, musicians showed larger negative lag-1 autocorrelations than non-musicians during the synchronization task, indicative of greater error correction (Wing and Kristofferson, 1973; Sowiński and Dalla Bella, 2013). Finally, recurrence quantification analyses revealed more repetitive, patterned, and mathematically stable synchronization behavior for non-musicians than for musicians. These findings are consistent with postural sway differences between patients with Parkinson’s Disease (PD) and control participants (Schmit et al., 2006), and between track athletes and expert ballet dancers (Schmit et al., 2005). In both studies, the less skilled group (i.e., PD patients and track athletes) demonstrated more repetitive and patterned postural sway; our findings show a similar pattern of behavior in non-musicians during a synchronization task. The decreased noise observed in non-musicians’ synchronization behavior is consistent with a dynamical systems perspective that a system with less noise should also be less flexible (Schmit et al., 2005). Indeed, we provide evidence that non-musicians synchronize less flexibly across rates than musicians.

The current experiments also examined the contributions of peripheral timing mechanisms to spontaneous rates. In Experiment 1, wrist width and piano performance SPR significantly predicted SPR: Participants with wider wrists tended to have slower SPRs. In Experiment 2, no relationship was observed between participants’ wrist widths and SPRs. These conflicting results may have arisen due to differences in the tapping task across the two experiments. In the first experiment, participants tapped on a piano keyboard without arm support. In the second experiment, participants tapped on a FSR positioned on a table where they could rest their arm. Therefore, while the tapping motion in the first experiment likely came primarily from the wrist (Dennerlein et al., 2007), this may not have been true for the second experiment. Further research is needed to investigate how changes in movement execution influence peripheral contributions to spontaneous rates.

One limitation of the findings is their use of a small number of simple melodies to ensure musicians’ and non-musicians’ equivalent familiarity with the stimulus materials. Future studies should investigate a wider range of complex melodies to see whether the same results hold. Additionally, maximal rate measures from the second experiment indicated that some participants may not have tapped at their true maximal rates. The maximal rate task may have reflected cognitive as well as motor constraints. Future measurements of maximal rates may utilize isochronous tasks without the presence of auditory feedback to get a purer measure of biomechanical constraints.

In sum, both spontaneous rates and musical training modulated the degree to which individuals flexibly performed a synchronization task across rates. Consistent with the view that spontaneous rates act as an attractor in well-learned tasks, both musicians and non-musicians show biases in synchronization toward a natural frequency that cannot be explained solely by musical training or biomechanical hand differences. Finally, we show that musical training enhances performers’ flexibility in moving away from this attractor. This enhanced flexibility is characterized by greater engagement of error correction mechanisms as well as less patterned (noisier) synchronization behaviors that may facilitate rate adaptation. Predictors of rate flexibility included tapping regularity at the spontaneous rate in the absence of a pacing cue; individual differences in this temporal regularity predicted rate flexibility for musicians, but not for non-musicians. Future research may investigate the neural mechanisms underlying musicians’ enhanced flexibility around spontaneous rates, with an emphasis on treating musical training as a continuum.

## Ethics Statement

This study was carried out in accordance with the recommendations of the McGill University Policy on the Ethical Conduct of Research Involving Human Participants and the Tri-Council Policy Statement: Ethical Conduct for Research Involving Humans, McGill University Research Ethics Board with written informed consent from all subjects. All subjects gave written informed consent in accordance with the Declaration of Helsinki. The protocol was reviewed by the McGill University Research Ethics Board.

## Author Contributions

All authors contributed to the design and implementation of the experiments. RS performed the data analysis and wrote drafts of the manuscript. AZ contributed to data analysis and editing the manuscript. CP contributed to writing and editing the manuscript.

## Conflict of Interest Statement

The authors declare that the research was conducted in the absence of any commercial or financial relationships that could be construed as a potential conflict of interest.
