# Supplementary material for: Tapping Into Rate Flexibility: Musical Training Facilitates Synchronization Around Spontaneous Production Rates
Source: Front Psychol. 2018 Apr 6;9:458. doi: 10.3389/fpsyg.2018.00458 (PMC5897499; doi:10.3389/fpsyg.2018.00458)
Supplement: Supplementary file 1 [file Presentation_1.pdf]

Supplementary Material

Tapping into Rate Flexibility: Musical Training Facilitates Synchronization around Spontaneous Production Rates

Rebecca Scheurich[1], Anna Zamm[1], and Caroline Palmer[1]*

[1]Sequence Production Laboratory, Department of Psychology, McGill University, Montreal, QC, Canada

***Correspondence:**
Dr. Caroline Palmer
caroline.palmer@mcgill.ca

1 Supplementary Figures

The time from the start of the tap on the force sensitive resistor (FSR) to the start of the MIDI signal sent from the Arduino was measured with a Tektronix TDS 2002 oscilloscope. Data was recorded from 100 taps on the FSR and showed that this duration averaged 1.0 millisecond (SD = 0.035; see Supplementary Figure 1).

A MIDI signal was generated from the Arduino in response to a tap on the FSR and was sent via an M-Audio UNO MIDI-USB connector to a Linux computer running FTAP (Finney, 2001). FTAP processed the incoming MIDI signal from the Arduino and sent an outgoing MIDI signal. The time from the start of the MIDI signal sent from the Arduino to the start of the MIDI signal sent from the computer was measured with a Tektronix TDS 2002 oscilloscope. Data was recorded from 100 taps on the FSR and showed that this duration averaged 1.4 milliseconds (SD = 0.13; see Supplementary Figure 2).

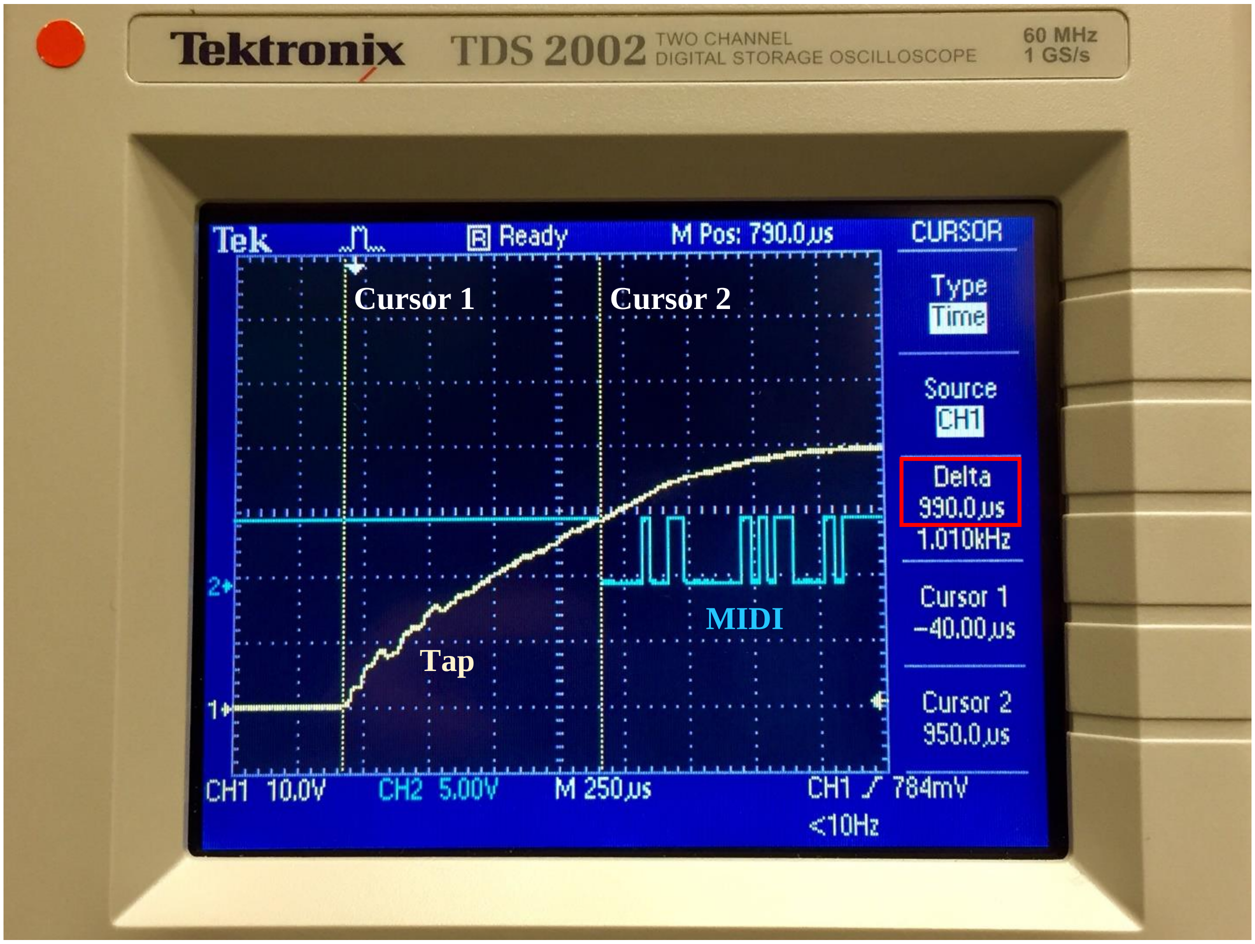

Supplementary Figure 1. Sample trial measuring the time from the start of the tap on the FSR (yellow trace) to the time at which the MIDI signal (blue trace) is sent from the Arduino. Cursor 1 is aligned to the start of the tap on the FSR, and cursor 2 is aligned to the start of the MIDI signal sent from the Arduino. Delta (0.99 milliseconds) calculates the difference between cursors 1 and 2.

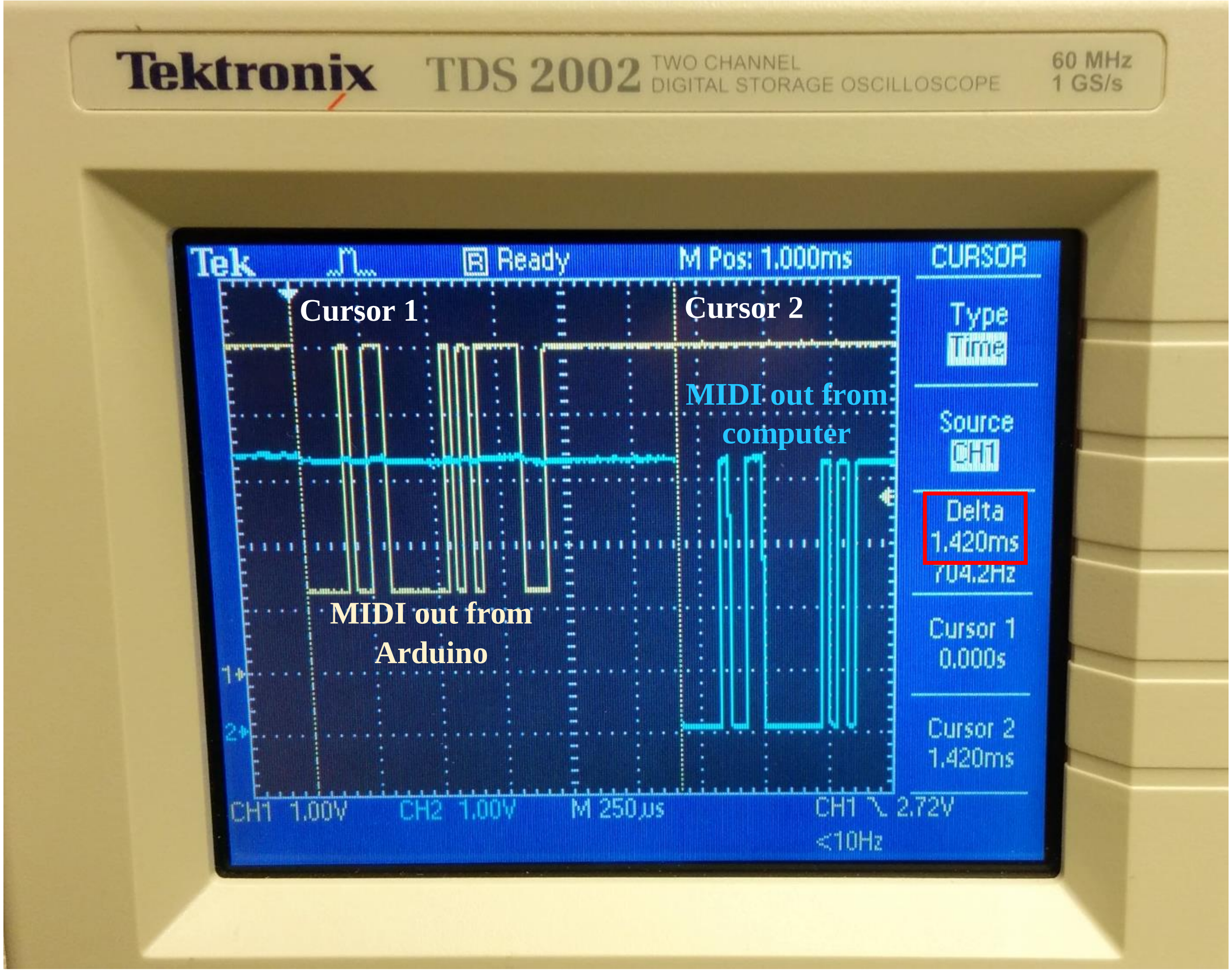

Supplementary Figure 2. Sample trial measuring the time from the start of the MIDI signal sent from the Arduino (yellow trace) to the time of the start of the MIDI signal sent from the computer (blue trace). Cursor 1 is aligned to the start of the MIDI signal sent from the Arduino, and cursor 2 is aligned to the start of the MIDI signal sent from the computer. Delta (1.42 milliseconds) calculates the difference between the signal onsets marked by cursors 1 and 2.
